# Glenohumeral cartilage lesions: reviewing diagnostic imaging strategies and preoperative evaluation

**DOI:** 10.1590/0100-3984.2025.0132

**Published:** 2026-09-08

**Authors:** Dâmaris Versiani Caldeira Gonçalves, Marco Aurélio Soato Ratti, Júlio Brandão Guimarães, Alípio Gomes Ormond Filho, Luiz Henrique Boraschi Vieira Ribas

**Affiliations:** 1 Department of Musculoskeletal Radiology, Fleury Medicina e Saúde, São Paulo, SP, Brazil; 2 Department of Radiology and Biomedical Imaging, University of California, San Francisco, CA, USA; 3 Department of Orthopedic Surgery, Shoulder and Elbow Unit, Grupo Vita, São Paulo, SP, Brazil

**Keywords:** Shoulder joint, Osteoarthritis, Cartilage, articular, Diagnostic imaging, Preoperative care., Articulação do ombro, Osteoartrite, Cartilagem articular, Diagnóstico por imagem, Cuidados pré-operatórios.

## Abstract

Glenohumeral cartilage lesions represent a significant challenge in both diagnosis and management due to the com-plex anatomy of the joint and the variability in lesion presentation. In this review, we elucidate the anatomical consid-erations of the glenohumeral joint, discuss multimodal diagnostic imaging strategies, and highlight imaging features critical for the effective management of focal and diffuse cartilage lesions in the glenohumeral joint, especially in the preoperative context. We also present the role of advanced magnetic resonance imaging techniques and the crucial parameters that radiologists must report in preoperative planning, in order to optimize outcomes in clinical practice.

## Abstract (pt)

Lesões da cartilagem glenoumeral representam um desafio tanto para o diagnóstico quanto para o seu apropriado manejo, devido à complexidade anatômica articular e à variabilidade de apresentação dessas lesões. Nesta revisão, abordamos a avaliação por imagem dessas lesões, destacando aspectos imaginológicos importantes para o manejo de lesões condrais glenoumerais focais e difusas, especialmente no contexto pré-operatório. Também trazemos in-formações sobre técnicas de imagem avançadas e parâmetros importantes de serem relatados no laudo dos exames para a obtenção de melhores resultados clínicos.

## INTRODUCTION

The glenohumeral joint is formed by the articula-tion between the shallow glenoid cavity of the scapula and the hemispherical head of the humerus in a configuration that allows great range of movement - as it is the most mobile joint in the human body. Its inherent instability derives primarily from the discrepancy between the articular surfaces of these joint components, because the humeral head is much larger than the glenoid cavity^**(^1^,^2^)**^.

Variations in glenoid morphology, such as hypoplasia and retroversion, may predispose the joint to instabil-ity^**(^3^,^4^)**^. Soft tissue stabilizers, both static and dynamic, compensate for that mismatch. When those structures fail, internal imbalances may lead to cartilage damage^**(^1^,^5^)**^.

Multiple other factors such as exercise and occupa-tion, as well as aging, inflammation, obesity and genet-ics, accelerate cartilage degeneration^**(^6^-^8^)**^. Glenohumeral cartilage lesions lead to progressive joint degeneration (osteoarthritis), which has substantial impact on affected individuals; in severe cases, osteoarthritis requires surgery for joint replacement^**(^7^-^9^)**^.

This review discusses the intricate anatomy of the glenohumeral joint and the spectrum of chondral le-sions. We also highlight the latest diagnostic imaging strategies that can contribute to patient management and improve the quality of radiologic reports, especially in the preoperative setting.

## GLENOHUMERAL JOINT STABILIZERS

Glenohumeral stability is maintained by static and dynamic stabilizers, which work synergistically to preserve joint congruency, as well as to prevent excessive transla-tion and rotation^**(^1^,^2^)**^.

Static stabilizers include the glenoid labrum, the joint capsule and the glenohumeral ligaments. The joint capsule is thick and strong, attaching to the humeral neck and scapula just beyond the glenoid labrum. How-ever, it is lax inferiorly, forming a larger axillary recess ^**(^1^,^2^)**^. It is lined internally by the synovial membrane and displays focal thickened bands anteriorly-prop-erly referred to as the superior, middle, and inferior glenohumeral ligaments-which provide additional resistance against anterior and inferior translation^**(^1^,^10^)**^. The coracohumeral ligament, extending from the cora-coid process to the humeral tuberosities, provides ad-ditional support superiorly, limiting joint flexion and extension. The joint capsule itself is also a significant static stabilizer, particularly in terms of limiting the extremes of motion^**(^1^,^2^,^5^)**^.

**Figure 1:**
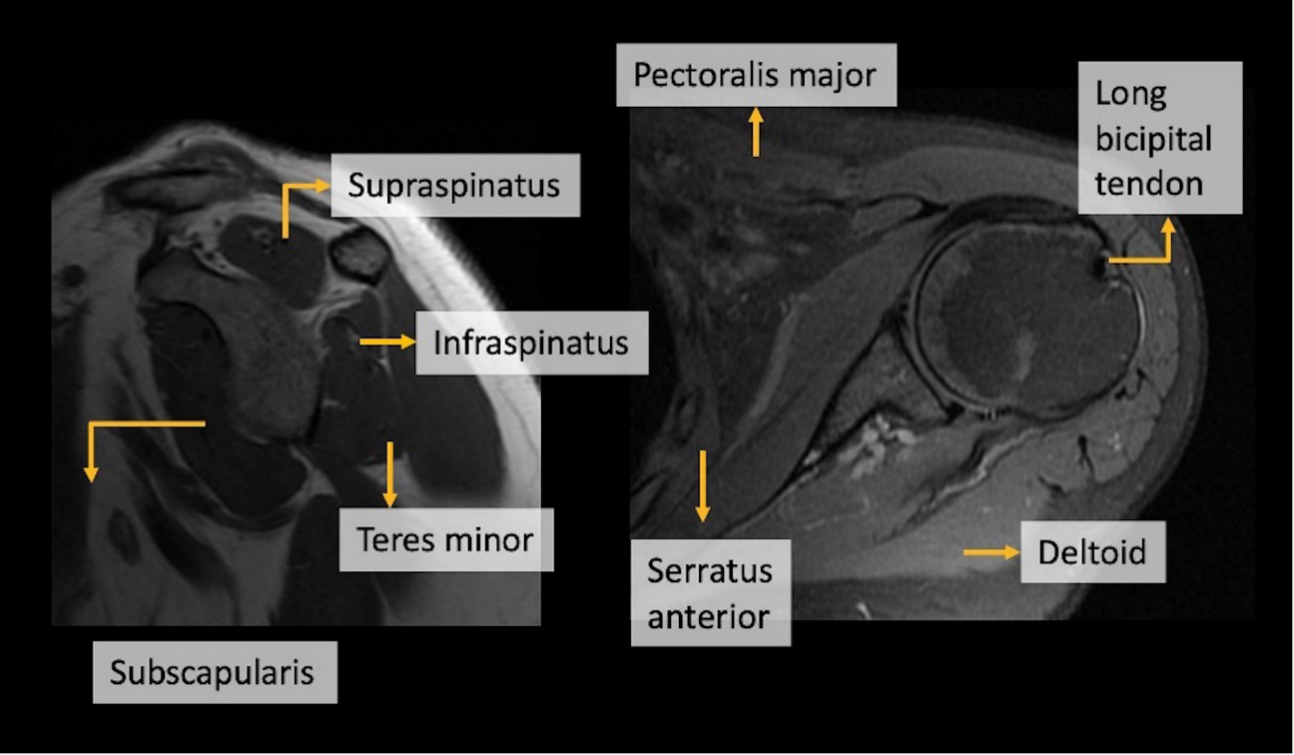
CT scans of the muscles surrounding the shoulder joint. The rotator cuff muscles provide compressive forces that center the humeral head within the glenoid. The long head of the biceps brachii, deltoid, and periscapular muscles (pectoralis major and serratus anterior) contribute to dynamic joint stability by balanc-ing forces during shoulder movements.

Dynamic stabilization of the glenohumeral joint is primarily attributed to the rotator cuff muscles (supraspinatus, infraspinatus, teres minor, and sub-scapularis), which provide compressive forces that center the humeral head within the glenoid (Figure 1). The long head of the biceps brachii, deltoid, and other periscapular muscles also contribute to dynamic joint stability by balancing forces during shoulder move-ments. Effective coordination among these muscles is essential for maintaining shoulder stability and preventing pathological lesions^**(^1^,^11^,^12^)**^.

## GLENOHUMERAL CHONDRAL LESIONS

**Figure 2:**
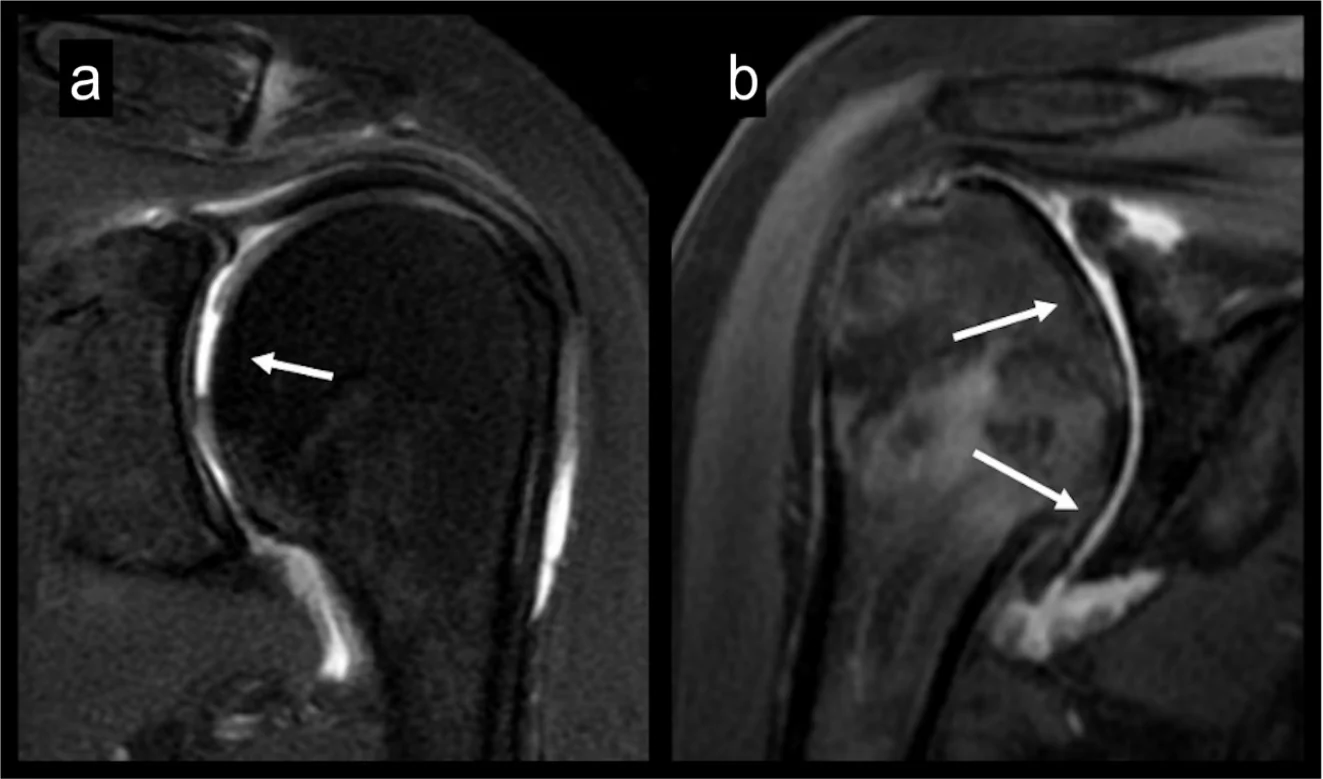
MRI findings of focal versus dif-fuse glenohumeral chondral lesions. (**A**) Fo-cal chondral lesion (arrow): typically seen in younger, physically active patients with acute-onset shoulder pain. These lesions are less common and often associated with trauma, joint instability, or mechani-cal overload. (**B**) Diffuse chondral lesion (arrows): commonly found in older patients with chronic symptoms, representing gle-nohumeral osteoarthritis.

Glenohumeral chondral lesions are categorized as focal or diffuse, each with distinct etiologies, clinical implications, and management strategies. Focal lesions are more commonly associated with acute trauma or re-petitive microtrauma, as illustrated in Figure 2A, whereas diffuse lesions often result from degenerative processes such as osteoarthritis ^**(^6^,^13^-^16^)**^ , as depicted in Figure 2B).

### Focal lesions

**Figure 3:**
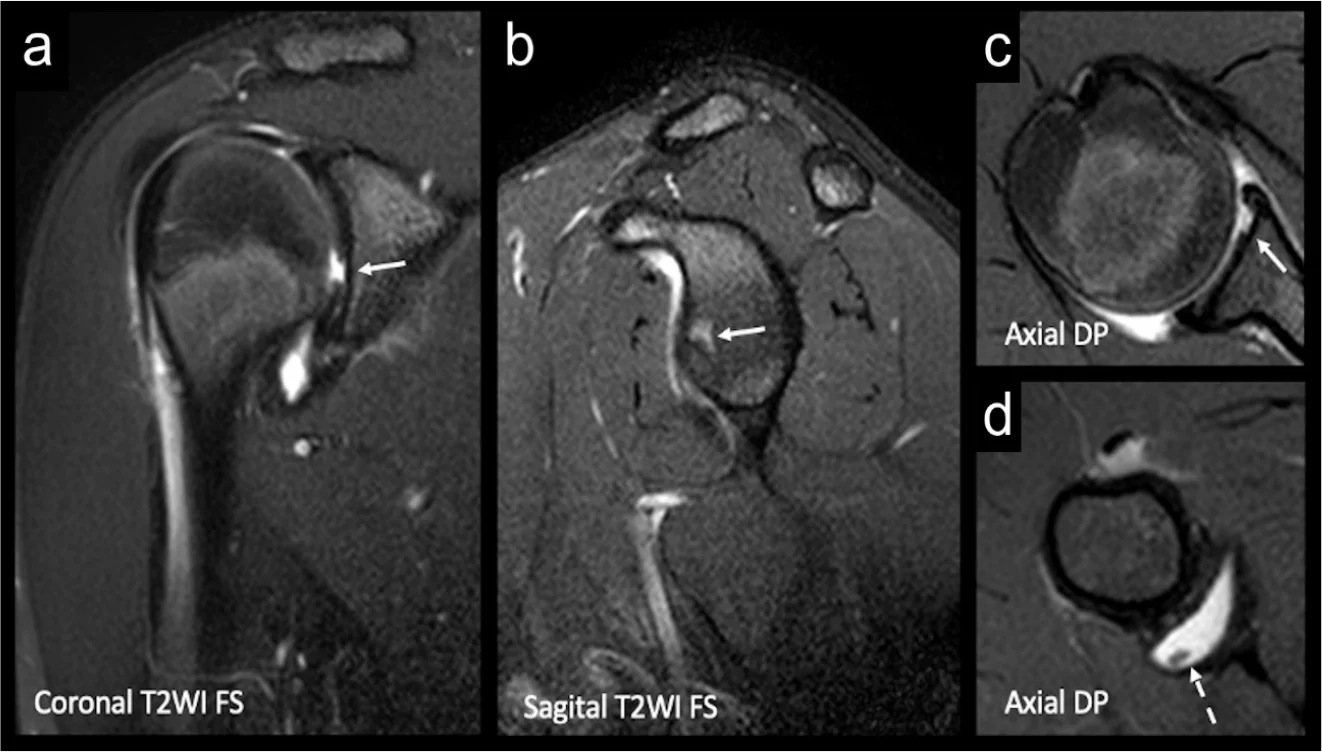
A 21-year-old male who present-ed with right shoulder pain after strenu-ous physical activity. (**A-C**) Fat-saturated T2-weighted MRI sequences showing a deep focal chondral lesion in the anterior region of the glenoid (arrows). (**D**) A chon-dral fragment was detected in the axillary recess (dashed arrow).

Focal chondral lesions are localized cartilage defects that can appear as fissures, flaps, or full-thickness loss (Figure 3). The reported incidence of symptomatic gleno-humeral chondral defects ranges from 5% to 17%^**(^15^)**^. Their etiology comprises previous surgical and traumatic condi-tions, including iatrogenic damage during arthroscopy, repetitive microtrauma and overuse. Younger individuals and athletes are the most commonly affected^**(^6^,^13^,^15^-^17^)**^.

Clinical manifestations of focal chondral lesions are nonspecific, including pain, clicking and locking, and may overlap with other shoulder pathologies. Therefore, arthroscopy remains the gold standard for their diagno-sis^**(^13^,^15^,^17^)**^. Because either or both articular surfaces may be involved, specificities regarding their isolated develop-ment are provided further below.

Glenoid chondral defects often coexistent with labral tears-the so-called “chondrolabral injuries”-related to mechanisms such as glenohumeral impaction, sublux-ation and dislocation, contributing to or resulting from joint instability. The spectrum of chondrolabral lesions encompasses glenoid labral articular disruption, glenoid articular rim divot, glenoid labral articular flap, and gle-noid labral articular teardrop, among other variants, all of them involving the anteroinferior segment of the glenoid labrum and the adjacent glenoid cartilage lining^**(^18^)**^.

In contrast, isolated nontraumatic humeral chon-dral lesions are less common. Factors such as humeral osteonecrosis, subchondral fractures and unstable os-teochondral fragments may be implicated in the genesis of humeral cartilage detachments^**(^19^)**^. Humeral cartilage lesions may also occur in overhead-throwing athletes, and their location in the posterosuperior humeral head aspect is frequently related to posterosuperior internal impingement; defects in superior humeral head segment may also be related to subacromial impingement^**(^20^,^21^)**^.

### Imaging diagnosis of focal chondral defects

**Figure 4:**
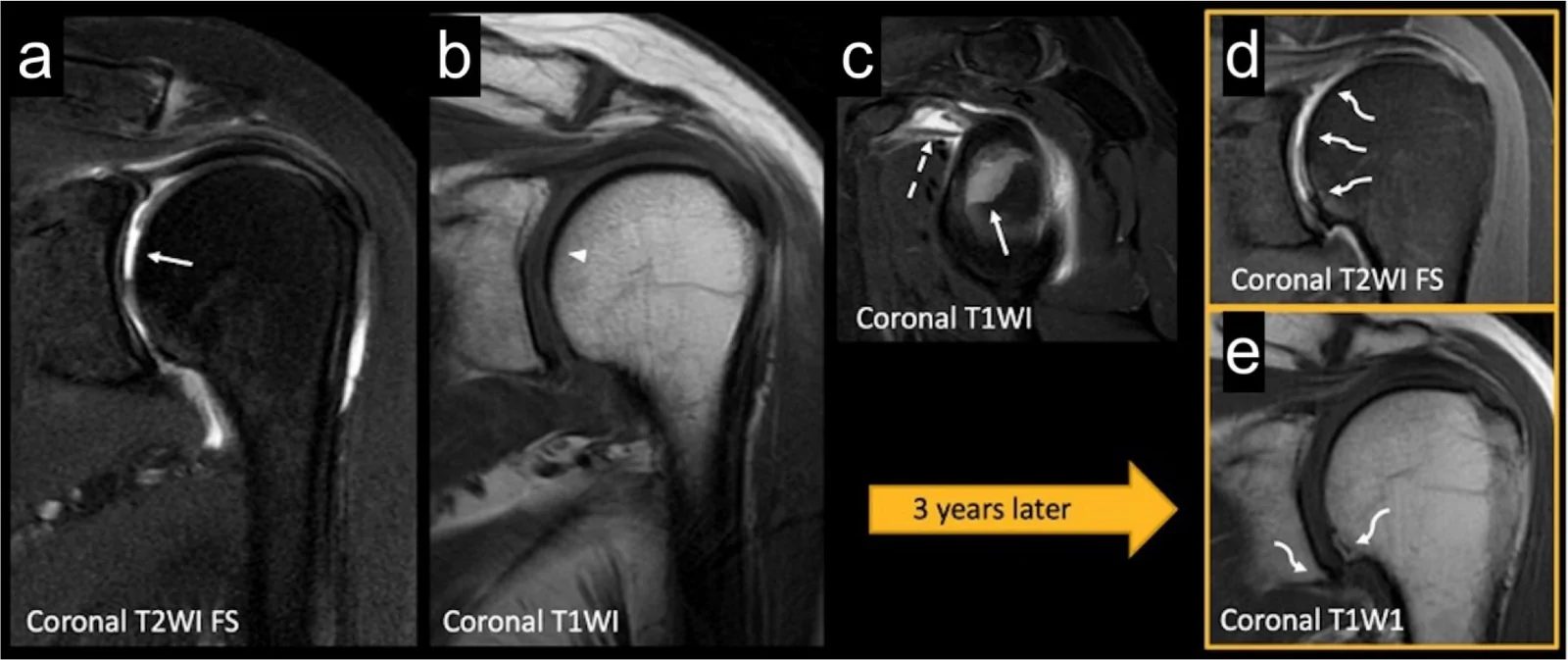
A 59-year-old female patient who presented with severe left shoulder pain after physical activity. (**A-C**) MRI scans showing a fo-cal chondral lesion in the humeral head (arrows), without subchondral signal alterations (arrowhead). A chondral fragment was detected in the subcoracoid recess (dashed arrow). (**D,E**) Three years later, diffuse chondral thinning and osteophytes were observed (curved arrows).

**Figure 5:**
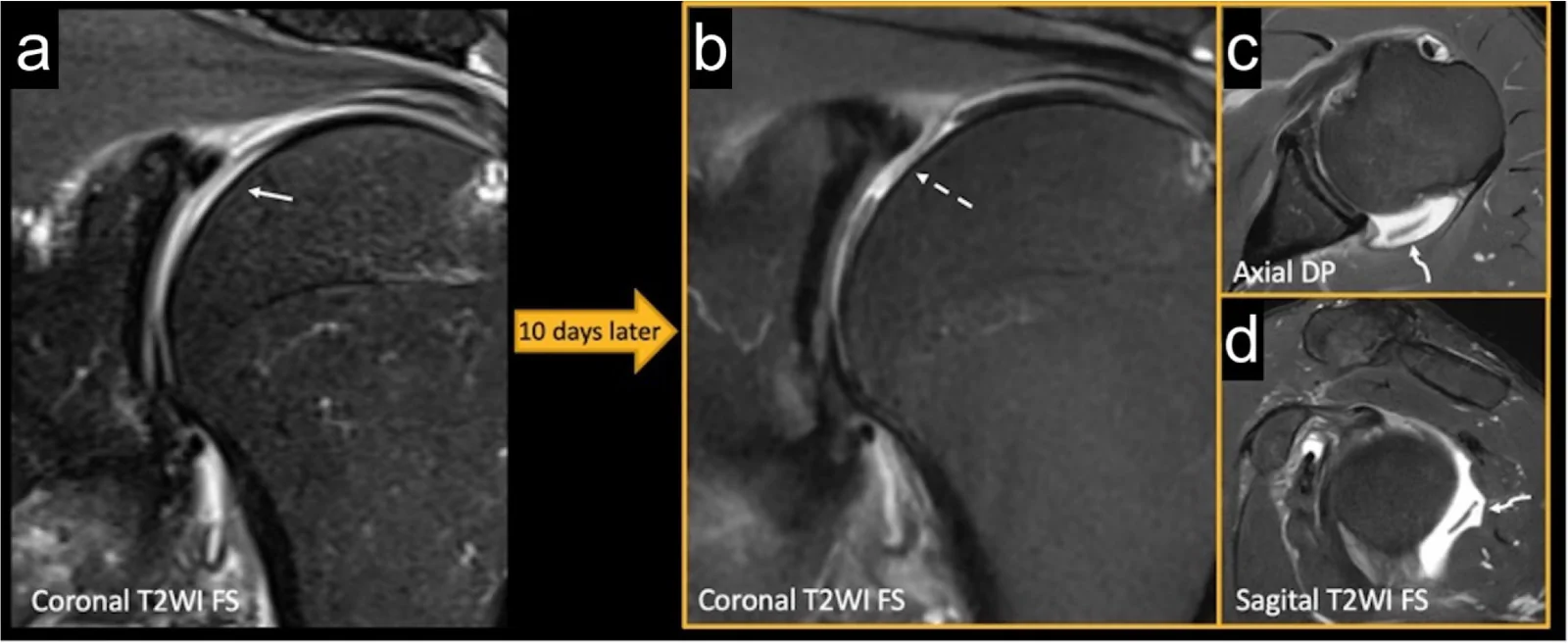
A 57-year-old male with acute pain after serving in a game of tennis. (**A**) Initial MRI scan showing a focal lesion suggesting chon-dral delamination in the humeral head (arrow), without subchondral signal alterations, and joint effusion with synovitis and pericapsular edema. (**B-D**) MRI scans acquired ten days later, after symptom worsening, highlighting the appearance of chondral erosion/detachment (dashed arrow) with a chondral flap displaced to the posterior joint recess (curved arrows).

Magnetic resonance imaging (MRI) and magnetic resonance arthrography (MRA) are crucial imaging mo-dalities for the diagnosis of focal cartilage lesions because they allow detailed visualization of cartilage, labrum, and other soft tissue structures^**(^22^)**^. However, the thin gleno-humeral cartilage lining and the curved articular contours make MRI less sensitive than arthroscopy for the detection of subtle chondral lesions^**(^23^,^24^)**^. Active searches for indi-rect findings such as subchondral bone marrow changes, synovitis, joint effusion, and intra-articular loose bodies may improve reading performance by radiologists^**(^25^)**^ , as illustrated in Figures 4 and 5.

Using scanners with field intensities ≤ 1.5 T, Momenzadeh et al.^**(^24^)**^ reported that MRI had an overall sensitivity and specificity of 90% for detecting humeral and glenoid cartilage lesions, although both indications varied according to the chondral lesion site. Similarly, MRA showed moderate performance in detecting gleno-humeral chondral defects, with a slightly higher sensitivity (approximately 77%) for detecting glenoid cartilage le-sions^**(^26^)**^. The use of MRA also improves the detection of labroligamentous and tendinous abnormalities, making it particularly valuable in cases of glenohumeral instability and sports-related injuries^**(^27^-^29^)**^.

Technological advances in MRI, with greater com-mercial availability of 3.0-T scanners, have improved image resolution, therefore reducing the need for MRA in clinical practice. However, the latter is still recommended for the investigation of shoulder instability in young pa-tients (particularly those who are overhead-throwing athletes), as well as of relapsing shoulder instability, in the assessment of postoperative labral tears, or in case of persistent clinical suspicion despite unremarkable MRI findings, given that MRA still demonstrates statistically better detection of shoulder lesions compared with con-ventional 3.0-T MRI, including anterior labral tears and superior labrum anterior to posterior tears^**(^30^,^31^)**^.

**Figure 6:**
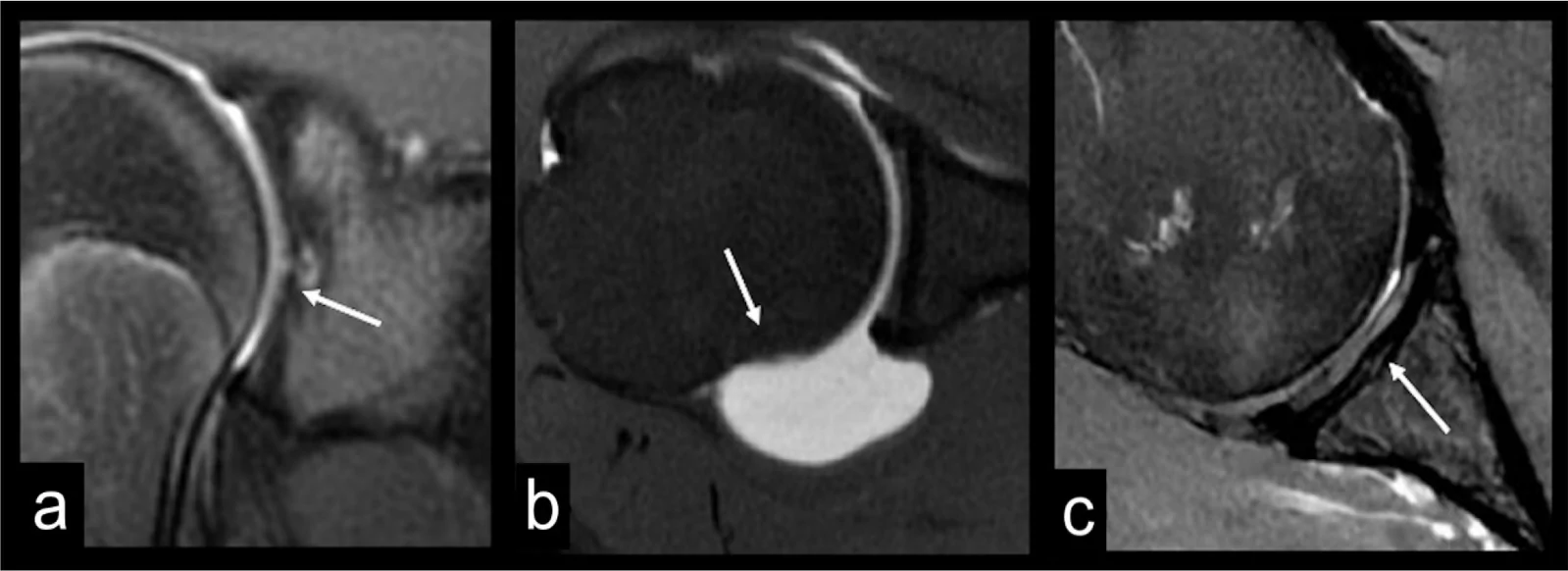
MRI findings of anatomical variants in the glenoid and humeral head. (**A**) Bare area of the glenoid: a normal focal articular defect located at the central and inferior aspect of the glenoid. (**B**) Bare area of the humeral head: a region between the posterior insertion of the joint capsule and the adjacent articular cartilage, which may mimic a Hill-Sachs impaction injury. (**C**) Tubercle of Assaki: a focal thicken-ing of the subchondral bone along the central aspect of the glenoid fossa.

Anatomical variations can pose diagnostic chal-lenges. For instance, the “bare areas” of the glenoid or humeral head (Figure 6), which are normal variants, may be misinterpreted as a pathological lesion if not carefully assessed^**(^10^,^32^)**^.

Advanced MRI techniques are emerging as nonin-vasive tools for shoulder cartilage assessment. Delayed Gadolinium-Enhanced MRI of Cartilage (dGEMRIC) assesses glycosaminoglycan depletion, an early indi-cator of cartilage degeneration^**(^33^-^35^)**^. This technique involves gadolinium contrast administration, allowing diffusion into cartilage over a period of time before T1 relaxation times are measured. Lower glycosaminogly-can levels correspond to higher gadolinium uptake, indicating early biochemical changes that precede structural cartilage damage^**(^33^,^35^)**^.

By focusing on structural integrity, as well as quan-tifying changes in cartilage water content and collagen organization, T2 mapping complements dGEMRIC. Signal changes on T2 mapping also precede gross mor-phological alterations visible on conventional MRI. Combining biochemical and structural assessments through dGEMRIC and T2 mapping offers a comprehensive approach to cartilage evaluation, particularly in early-stage osteoarthritis^**(^35^,^36^)**^.

Emerging modalities such as T1 mapping and sodium MRI show promise as alternatives for patients who can-not receive gadolinium-based agents, further enhancing diagnostic capabilities^**(^34^)**^.

Lastly, zero-echo time MRI of the shoulder is useful for complementary assessment of bone pathology that may be associated with glenohumeral chondral defects, avoiding the need for computed tomography (CT) and, consequently, exposure to radiation^**(^37^,^38^)**^.

### Management

The management of focal chondral lesions varies depending on the depth, extension and location of the chondral defects, as well as of any other shoulder lesions. Other factors, such as patient age, intensity of symptoms, impairment of quality of life, and physical activity level, also need to be considered.

Nonsurgical measures, including activity modifica-tion, physical therapy, oral nonsteroidal anti-inflammatory drugs (NSAIDs), and intra-articular injections (with corti-costeroids, local anesthetics or hyaluronic acid) are often the first-line treatments^**(^15^,^39^)**^.

For patients with refractory symptoms, joint-preserv-ing procedures such as arthroscopic debridement, micro-fracture, or cartilage transplantation may be indicated depending on the defect size and patient profile^**(^15^,^17^)**^. These procedures are aimed at relieving pain, restoring function, and prolonging joint integrity by stabilizing car-tilage surfaces and removing unstable flaps^**(^14^)**^. In cases of extensive chondral defects, resurfacing techniques, including nonbiological or biological interposition arthroplasty, may serve as alternatives to delay definitive arthroplasty^**(^39^,^40^)**^. Lastly, correction of coexistent le-sions of other shoulder structures, such as labral tears, must be included in the surgical planning and may even hasten the need for surgical treatment.

#### Diffuse lesions

**Figure 7:**
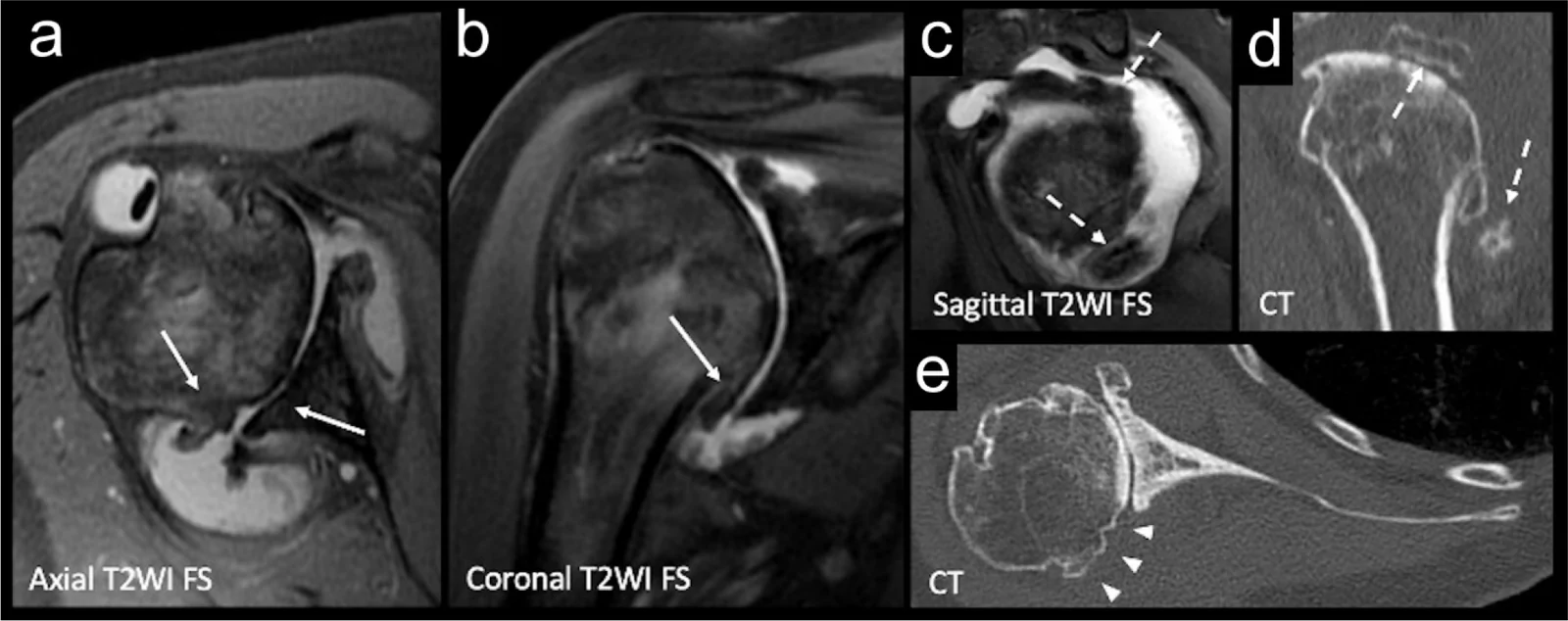
A 78-year-old woman with chronic right shoulder pain who was found to have advanced glenohumeral osteoarthritis. (**A-C**) Fat-saturated T2-weighted MRI sequences showing diffuse, irregular chondral thinning, posteroinferior marginal osteophytes (arrows), bone remodeling, and light subchondral edema. There are also joint effusion, synovitis and intra-articular ossifications (dashed arrows). (**D,E**) Those features were also observed on a concomitant CT scan of the shoulder. Notably, glenoid retroversion, humeral subluxation, and medialization were also observed (arrowheads).

**Figure 8:**
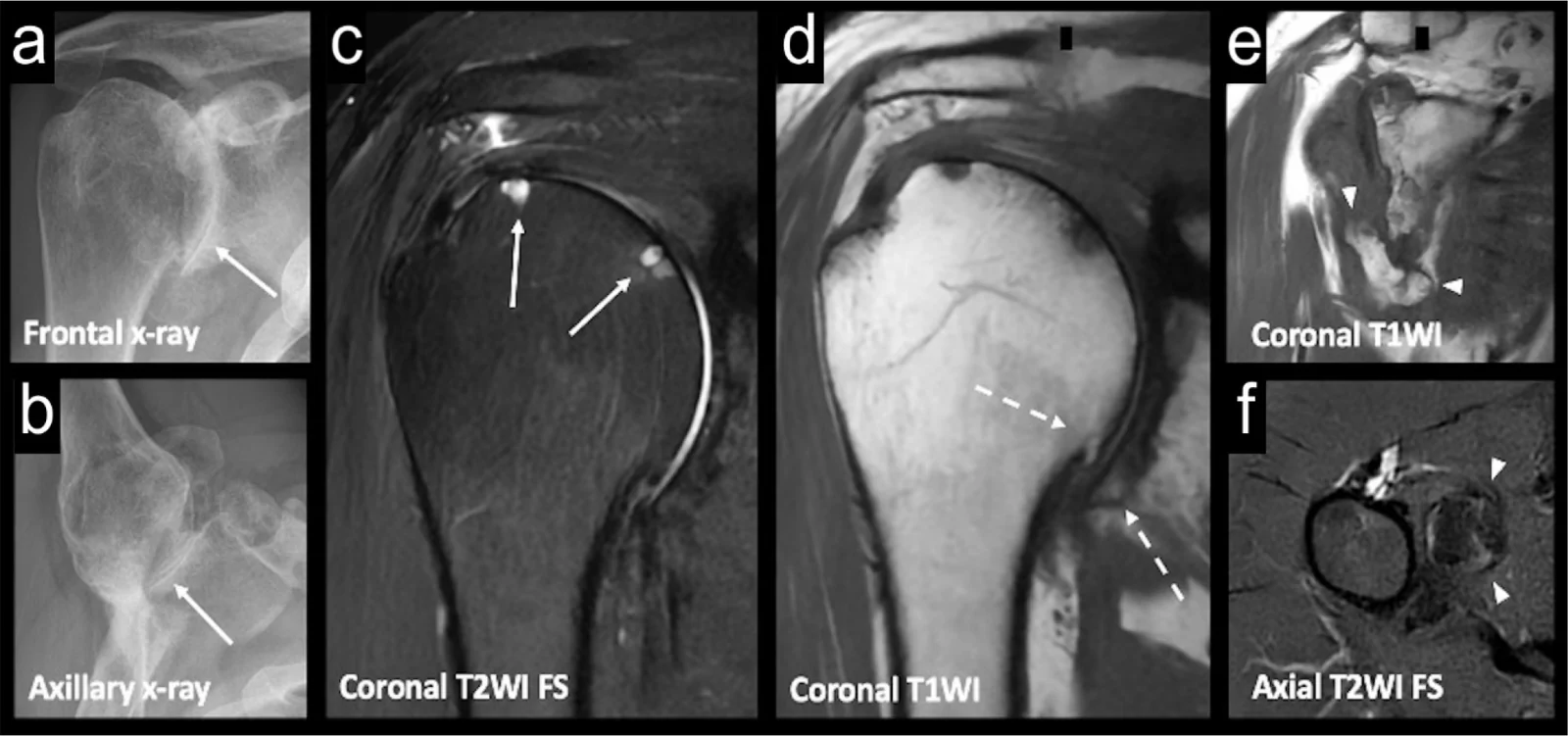
A 63-year-old male patient undergoing a post-traumatic MRI follow-up examination. (**A,B**) Frontal and axillary X-ray views show-ing joint space narrowing and bone remodeling, consistent with glenohumeral osteoarthritis. (**C-F**) MRI scans showing diffuse chondral thinning and reduced joint space, as well as areas of subchondral bone edema and cysts (arrows), together with marginal osteophytes (dashed arrows). Note the small glenohumeral joint effusion with a large, ossified intra-articular loose body (arrowhead).

T2WI, T2-weighted image; FS, fat-saturated; T1WI, T1-weighted image.

Diffuse lesions, such as those observed in glenohu-meral osteoarthritis, involve widespread degeneration of articular cartilage, typically affecting the humeral head and the glenoid, and may result in pain, loss of function, and diminished quality of life. Such lesions are character-ized by cartilage thinning, subchondral bone sclerosis, and osteophyte formation. Involvement of the soft tissues (synovium, joint capsule, ligaments, and rotator cuff tendons) may play a role in the progression of cartilage degeneration, either as a causative factor for cartilage loss or as its consequence^**(^41^)**^, as shown in Figures 7 and 8.

Unlike focal lesions, which are typically localized and associated with specific mechanical insults, diffuse lesions are commonly present in the context of glenohumeral osteoarthritis, either primary or secondary, affecting the articular surfaces of the humeral head and of the glenoid. In cases of primary glenohumeral osteoarthritis, diffuse lesions frequently occur in older populations and are closely linked to systemic factors such as inflammation, genetic predisposition, and hormonal imbalances^**(^8^,^41^)**^.

Although glenohumeral osteoarthritis is less com-mon than is knee or hip osteoarthritis, it has a con-siderable impact on affected individuals. Studies have shown that cartilage lesions are found in 5-15% of routine arthroscopy studies^**(^41^)**^. Population-level stud-ies estimate the prevalence to be as high as 32.8% in individuals over 60, though asymptomatic cases likely result in underreporting^**(^8^,^41^)**^. Gender differences are also notable, with a predominance of men in younger cohorts, whereas postmenopausal women predominate in older cohorts, possibly because of hormonal changes that influence cartilage metabolism^**(^41^)**^.

#### Etiology

The development of diffuse lesions in the gleno-humeral joint is multifactorial, involving primary and secondary mechanisms. These mechanisms highlight the complexity of osteoarthritis and the variety of factors that influence its progression^**(^6^,^7^,^41^,^42^)**^.

### Primary osteoarthritis

Primary osteoarthritis is mainly associated with aging and the natural degeneration of articular carti-lage without preceding trauma. As individuals age, the density of chondrocytes within the hyaline cartilage decreases, as does responsiveness to anabolic growth factors, which renders the cartilage more susceptible to wear and tear. Over time, the progressive loss of cartilage and subsequent mechanical imbalance lead to joint dysfunction. The prevalence of glenohumeral osteoarthritis rises sharply with age, affecting up to 27.5% of individuals over 80 years of age^**(^6^,^42^)**^.

Genetic predisposition also plays a significant role in primary osteoarthritis. Complex polygenic inheri-tance patterns contribute to variations in cartilage resilience and susceptibility to degenerative processes. In addition, systemic factors such as obesity exacerbate the mechanical and biochemical stress on cartilage. Adipose tissue releases inflammatory cytokines that promote low-grade systemic inflammation, further accelerating cartilage breakdown^**(^6^,^32^,^42^)**^.

### Secondary osteoarthritis

Secondary osteoarthritis arises from identifiable causes that disrupt the biomechanics or structural in-tegrity of the joint.

Among the most common etiologies, post-traumatic shoulder disorders due to previous fractures and disloca-tions can directly damage the articular cartilage or alter joint alignment and lead to accelerated degeneration. Proximal humeral fractures and glenoid fractures, for instance, can result in chronic incongruence and joint instability, significantly increasing the risk of secondary osteoarthritis^**(^6^,^7^)**^. Likewise, in the setting of chronic gle-nohumeral instability, recurrent shoulder dislocation or subluxation imposes abnormal stress on the glenoid and humeral cartilage, contributing to chondral damage and, eventually, osteoarthritis. Studies indicate that individuals with recurrent dislocations are at a 10- to 20-times greater risk of developing radiological signs of osteoarthritis^**(^5^,^6^)**^.

Nontraumatic etiologies also play an important role in the development of secondary shoulder osteoarthritis. Autoimmune disorders such as rheumatoid arthritis and other inflammatory arthropathies (e.g., systemic lupus erythematosus and psoriatic arthritis) induce systemic inflammatory cascades that target synovial tissue and cartilage. Rheumatoid arthritis, in particular, frequently affects the glenohumeral joint, with bilateral involvement and determining central glenoid remodeling. Pain in these cases often correlates more strongly with inflammatory synovitis than with joint destruction itself^**(^2^,^6^)**^.

Septic arthritis caused by bacterial pathogens (e.g., *Staphylococcus aureus* or *Neisseria gonorrhoeae*) can rap-idly destroy joint cartilage if untreated. Early diagnosis and management are critical to prevent irreversible damage^**(^2^)**^.

Avascular necrosis (AVN) of the humeral head re-sults from an interruption of the blood supply, leading to localized bone collapse and secondary cartilage loss. The development of AVN can be post-traumatic or unrelated to trauma, due to prolonged corticosteroid therapy, alcohol abuse and systemic conditions such as sickle cell anemia. It is estimated that AVN accounts for 5% of all cases of secondary glenohumeral osteoarthritis^**(^6^,^41^)**^.

Extra-articular disorders can also generate joint im-balances and contribute to diffuse chondral damage. For instance, long-standing rotator cuff tears can lead to superior migration of the humeral head, causing abnor-mal contact with the glenoid and progressive cartilage deterioration. Secondary osteoarthritis is often accom-panied by remodeling of the coracoacromial arch and erosion of the superior glenoid, characteristic of cuff-tear arthropathy^**(^6^,^7^)**^. More rarely, neurological disorders, such as cervical syringomyelia and diabetic neuropathy, can impair joint proprioception and nociception, leading to unchecked joint destruction and instability^**(^6^)**^.

#### Diagnosis

The clinical diagnosis of glenohumeral osteoarthritis is limited because of the complex anatomy of the shoulder and the clinical similarities with primary differential diag-noses such as rotator cuff syndrome, subacromial bursitis, and adhesive capsulitis^**(^6^,^11^)**^. Thorough medical history taking remains essential, with the typical chief complaints including progressive pain, crepitation, reduced range of motion, and symptoms exacerbated by activity^**(^2^,^6^)**^. Clinical features that suggest a secondary etiology for glenohu-meral osteoarthritis must be actively sought: acute-onset pain along with exacerbated inflammatory changes favor the possibility of underlying inflammatory pathology, such as septic arthritis or microcrystalline disorders, whereas chronic pain accompanied by morning stiffness is a feature of rheumatoid arthritis^**(^2^,^6^)**^.

The diagnostic approach to diffuse lesions includes a combination of plain radiography, CT, and MRI.

Radiography is usually the first imaging modality required for evaluating glenohumeral lesions. Although it has limitations in detecting early cartilage damage, it remains essential for identifying structural bony changes, such as joint space narrowing, osteophyte formation, and subchondral sclerosis^**(^6^,^43^,^44^)**^. The radiographic shoulder series in a nontraumatic setting includes anteroposterior (AP) view in internal rotation, as well as true AP, axillary, and lateral Y views^**(^45^,^46^)**^. The most common radiographic abnormality is the presence of osteophytes, which can be an earlier marker of degeneration, making osteophyte identification clinically relevant^**(^44^,^46^)**^. The axillary view is particularly useful for visualizing joint space narrow-ing, which may be obscured in standard AP views, and for detecting glenohumeral subluxation and asymmetric glenoid remodeling^**(^44^)**^.

**Figure 9:**
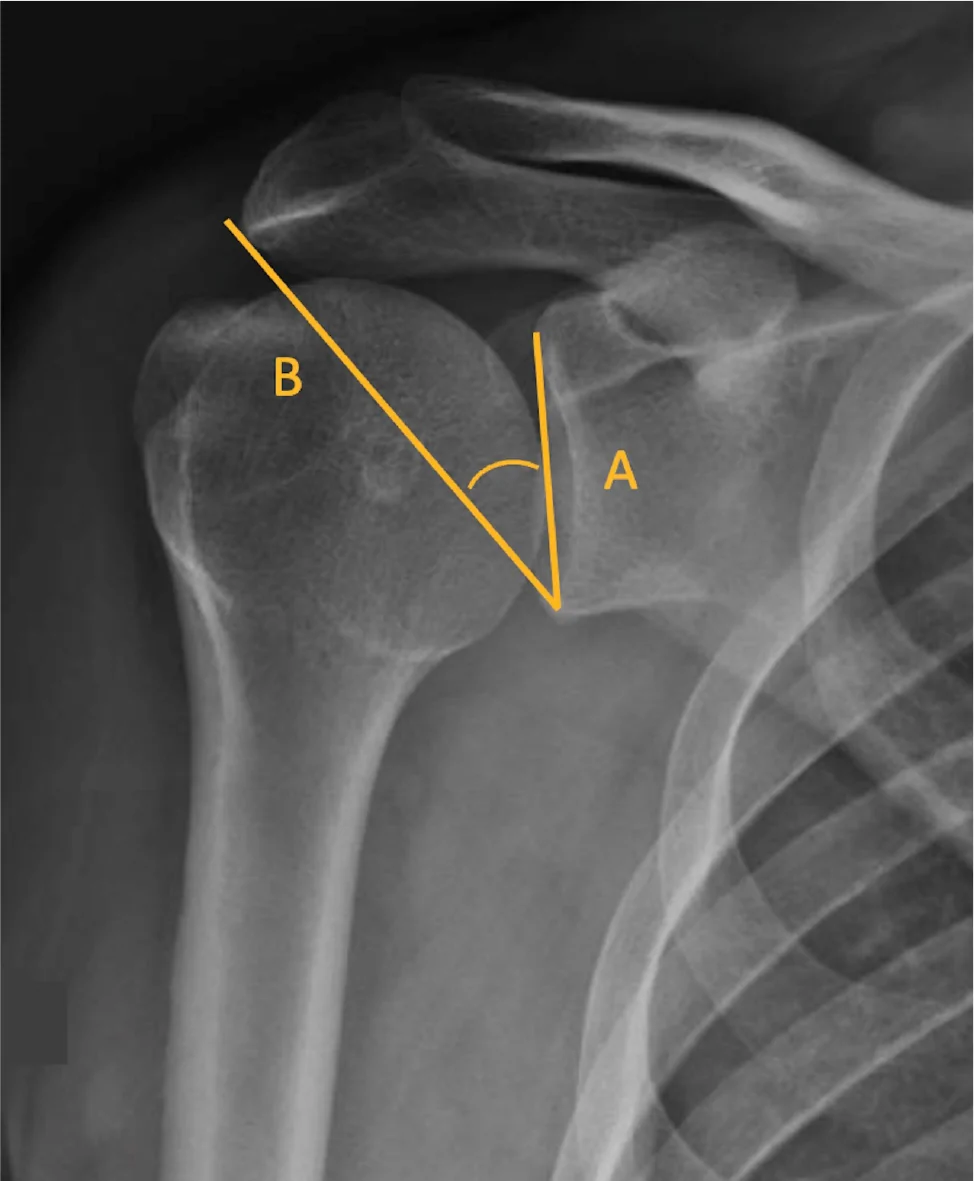
An AP X-ray view of the shoulder depicting the CSA. A: Line from the superior to inferior pole of the glenoid cavity. B: Line from the inferior border of the glenoid to the most infero-lateral border of the acromion.

**Figure 10:**
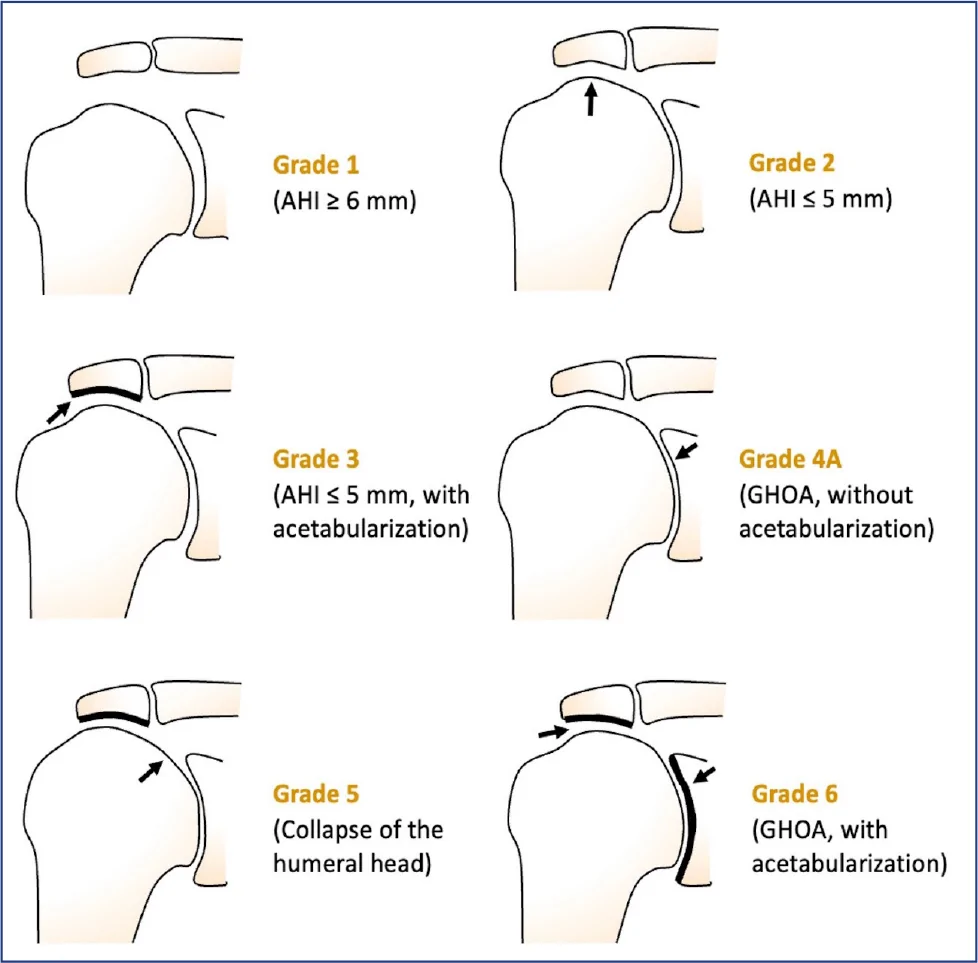
The Hamada classification of massive rotator cuff tears. This classification correlates the degree of rotator cuff pathology with radiological changes in the shoulder. Starting from an appar-ently normal radiograph (grade 1), the earliest change is the reduc-tion of the AHI (grade 2). As the rotator-cuff lesions and joint degen-eration progress, remodeling of the lower margin of the acromion (“acetabularization”) can be observed (grade 3) or the glenohumeral joint space decreases (grade 4). Grades 5 and 6 are the late stages, with a combination of subacromial and glenohumeral remodeling.

The Samilson-Prieto classification is commonly used in order to grade glenohumeral osteoarthritis based on the presence and size of osteophytes on AP views of the shoulder^**(^47^)**^, as detailed in Table 1. Another important radiographic parameter, albeit not diagnos-tic, is the critical shoulder angle (CSA), as depicted in Figure 9, which is associated with degeneration risk^**(^48^)**^: an increased CSA (> 35°) correlates with rotator cuff pathology, whereas a decreased CSA (< 30°) is linked to concentric osteoarthritis. The Hamada grading system helps describe radiographic progression of glenohu-meral degenerative changes related to chronic rotator cuff tears, ranging from acromiohumeral interval (AHI) narrowing to humeral head collapse and cuff-tear ar-thropathy^**(^49^,^50^)**^, as shown in Figure 10.

**Table 1:** Samilson-Prieto classification.

Grade	Osteophyte diameter
1	< 3 mm
2	3-7 mm
3	7 mm

Adapted from Elsharkawi et al.**^(^47^)^**.

GHOA, glenohumeral osteoarthritis.

In the assessment of glenoid morphology, bone stock, and preoperative surgical planning, CT plays a crucial role. Compared with conventional radiography, CT provides superior cortical bone definition and is particularly useful for evaluating glenoid version, subluxation of the humeral head, and the extent of glenoid bone loss in detail^**(^43^,^51^)**^. Three-dimensional (3D) CT reconstructions have become fundamental for surgical planning, allowing detailed spatial analysis and improving intraoperative precision. It is especially useful for preoperative planning in shoulder arthroplasty, enabling the best quantification of glenoid bone stock^**(^43^,^44^,^52^)**^.Finally, MRI is useful in the context of glenohumeral osteoarthritis for assessing the soft tis-sues in the shoulder, providing a detailed evaluation of the articular cartilage, labrum, shoulder muscles and rotator cuff tendons^**(^53^)**^. Preoperative knowledge on the status of these structures is fundamental for determining the appropriate surgical technique, as explained below.

#### Management

Treatment for diffuse lesions spans from nonop-erative approaches to advanced surgical interventions, depending on the severity of joint degeneration and patient-related factors. Similarly to what is done in the setting of focal chondral lesions, the management of glenohumeral osteoarthritis is initially conservative, relying on physical therapy for strengthening the rotator cuff and scapular stabilizers, as well as on oral NSAIDs and intra-articular injections to provide symptomatic relief^**(^6^,^17^,^41^)**^. These strategies have proven to be useful for symptom control and for the restoration of function, at least in the short-term^**(^54^,^55^)**^.

**Figure 11:**
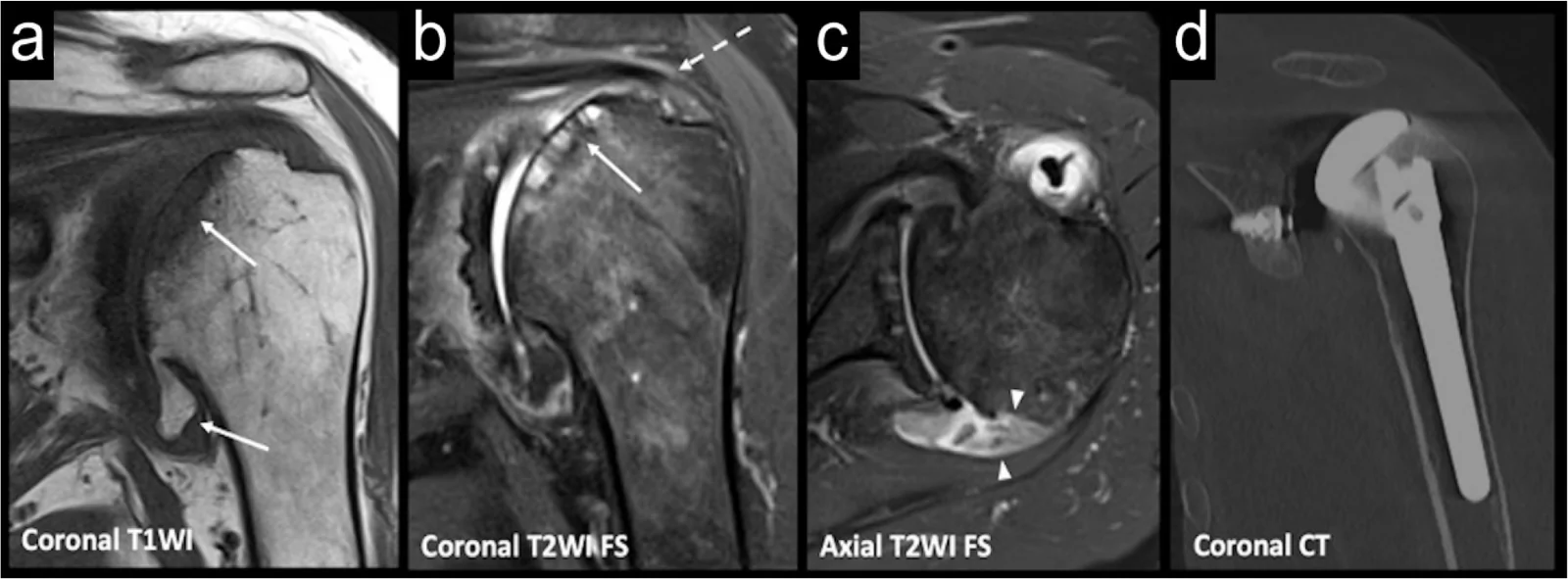
A 68-year-old female patient with advanced glenohumeral osteoarthritis. (**A-C**) MRI scans showing diffuse chondral thinning, subchondral bone cysts, and prominent marginal osteophytes (arrows). Note the small joint effusion with synovitis (arrowheads). The medical team opted for surgical treatment. Despite the presence of discrete partial tears, the rotator cuff tendons were mostly preserved (dashed arrows), leading to the decision to perform TSA. (**D**) Post-TSA CT scan.

T1WI, T1-weighted image; T2WI, T2-weighted image; FS, fat-saturated.

In refractory cases, surgical options for glenohumeral osteoarthritis are less extensive, because arthroscopic debridement may be effective only in early-stage lesions, making it inadequate for addressing advanced disease. Total shoulder arthroplasty (TSA) is the gold standard for end-stage glenohumeral osteoarthritis (Figure 11), benefiting from modern prosthetic designs, addressing glenoid bone loss, improving patient outcomes, and deliv-ering better postprocedure results than does hemiarthro-plasty^**(^9^,^43^)**^. Reverse shoulder arthroplasty is preferred in patients with severe rotator cuff dysfunction or significant joint deformities^**(^56^)**^.

#### Preoperative imaging assessment

Accurate preoperative imaging assessment is vital for planning surgical interventions in glenohumeral cartilage lesions. It requires detailed analysis of glenoid morphology, bone stock, version, and soft tissue status, to optimize surgical outcomes. Must-report findings are organized in Table 2 and discussed below.

**Table 2:** Preoperative assessment for glenohumeral joint osteoarthritis: what to report.

Aspect	Means of assessment
Glenoid morphology	• Anteversion/retroversion: measure angles based on the Friedman line
	• Measure glenoid bone stock
	• Evaluate glenoid dysplasia and glenoid degeneration: use the Walch classification
Humeral position in relation to glenoid cavity	• Use the GHSI and humeral head medialization
Glenohumeral cartilage status	• Report chondral defects, subchondral edema/cystic modification, and bone remodeling
	Focal chondral lesions: provide the diameters of the lesion, ascertain the presence or absence of associated bone fragmentation, and search for intra-articular chondral loose bodies
Labral morphology	• Identify anatomic variations
	• Report any labral degeneration/rupture as a consequence of the degenerative process
Additional features	• Report irreparable rotator cuff tears, determining the Hamada classification and assessing fatty degeneration of the rotator cuff muscles
	• Evaluate the deltoid muscle
	• Report synovitis

### Glenoid version

**Figure 12:**
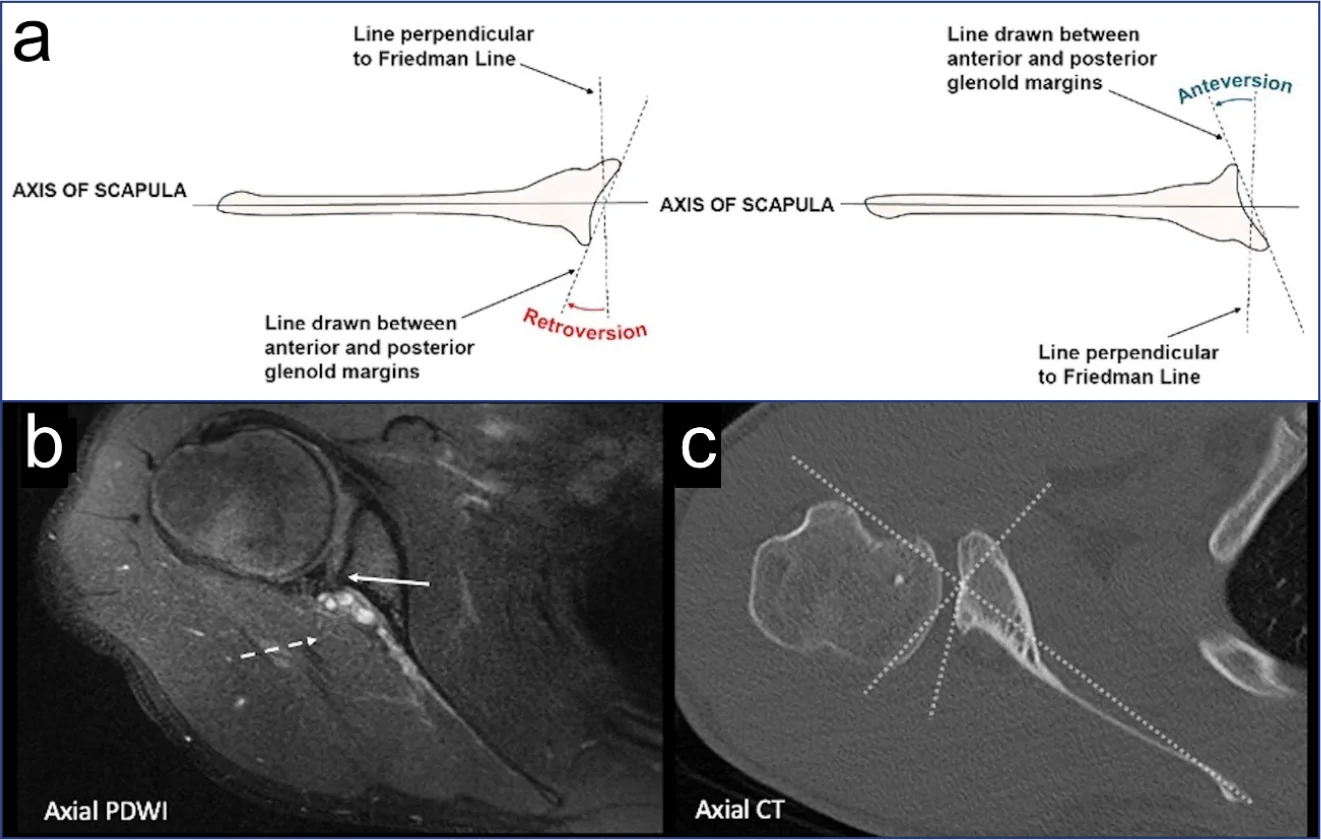
The Friedman method and practi-cal application. (**A**) The Friedman method in-volves drawing a line from the medial border of the scapula to the center of the glenoid (Friedman line), with a perpendicular refer-ence line representing neutral version. The angle between the glenoid articular surface andtheneutral linedefinestheversion. Agle-noid version of 0° ± 4° is considered normal; deviations beyond this range indicate retro-version or anteversion, both of which are as-sociated with increased prosthetic wear and joint instability. (**B,C**) A 23-year-old male who presented withchronicbilateral shoulder pain during exercise, without a history of trauma. (**B**) Fat-saturated T2-weighted MRIsequence showing a dysplasic glenoid, as well as a pos-terosuperior labral tear (arrow) withparalabral cysts (dashed arrow). (**C**) Estimated glenoid retroversion of 42°, as per the Friedman method, on axial CT, together with postero-superior bone remodeling.

Determining the glenoid version, defined as its orientation relative to the scapular axis, is crucial for surgical planning as it impacts implant positioning and joint stability^**(^43^)**^. The Friedman method, widely regarded as the most reliable approach, measures the glenoid version by using a scapular reference line to determine deviations from neutral alignment^**(^57^)**^, as detailed in Figure 12.

PDWI, proton density-weighted image.

The use of advanced imaging techniques, particu-larly 3D CT reconstruction, enhances the accuracy of the glenoid version measurement, providing clearer views of glenoid morphology and aiding in surgical planning. Variations such as significant retroversion (> 15°) or anteversion (> 5°) often necessitate special-ized implants or bone grafting to restore the natural joint alignment^**(^43^,^51^)**^.

### Bone stock

**Figure 13:**
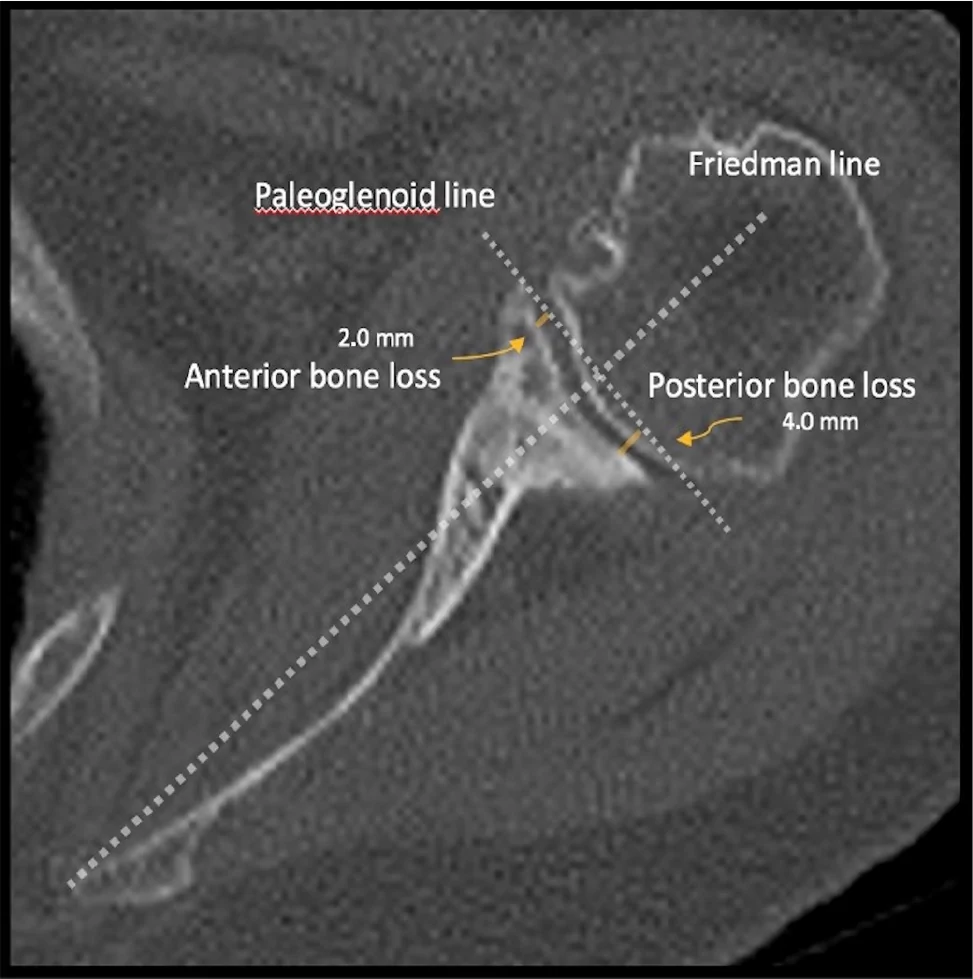
Bone stock assessment. In the axial plane, taking the Fried-man line as a primary reference, the paleoglenoid line must be drawn perpendicularly but placed at the most anterior extremity of the gle-noid. Glenoid bone resorption is measured in the anterior, central, and posterior parts of the glenoid, as the distance from this paleoglenoid line to the glenoid bone cortex in these sections. Osteophytes must be excluded from the quantification, so it is advised not to consider the first 5 mm from the anterior and posterior glenoid edges.

Bone stock assessment is crucial for ensuring the stability of the arthroplasty and longevity of the glenoid implant^**(^52^)**^. It enables qualitative and quantitative as-sessment of the glenoid bone resorption process in gle-nohumeral osteoarthritis, guiding orthopedic surgeons in determining whether there is need for glenoid augmen-tation in TSA planning for better postoperative results. Taking the Friedman line and the paleoglenoid line as references in the axial CT plane, bone stock should be measured in the anterior, central, and posterior portions of the glenoid cavity (Figure 13). The Pico method (“best-fit circle”) for measuring glenoid bone loss is more suitable in the setting of post-traumatic shoulder dislocations than in that of the preoperative period before elective surgery for glenohumeral osteoarthritis^**(^43^,^44^,^58^)**^.

### Glenohumeral subluxation assessment

**Figure 14:**
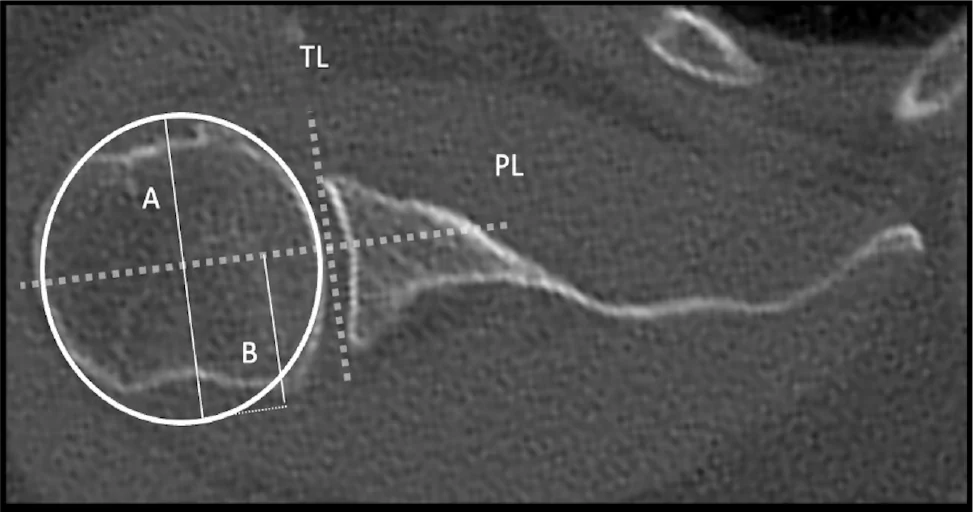
The GHSI, calculated as the ratio between the posterior width of the humeral head and the glenoid diameter. A GHSI greater than 0.55 indicates significant posterior subluxation, which corre-lates with glenoid retroversion and asymmetric cartilage thinning.

The glenohumeral subluxation index (GHSI) quan-tifies the posterior displacement of the humeral head relative to the glenoid, probably owing to capsuloliga-mentous laxity and labral lesions (Figure 14). The use of 3D imaging techniques improves the accuracy of GHSI determination by enabling precise segmentation of the humeral head and glenoid structures^**(^43^,^51^,^59^)**^.

### Humeral head medialization

**Figure 15:**
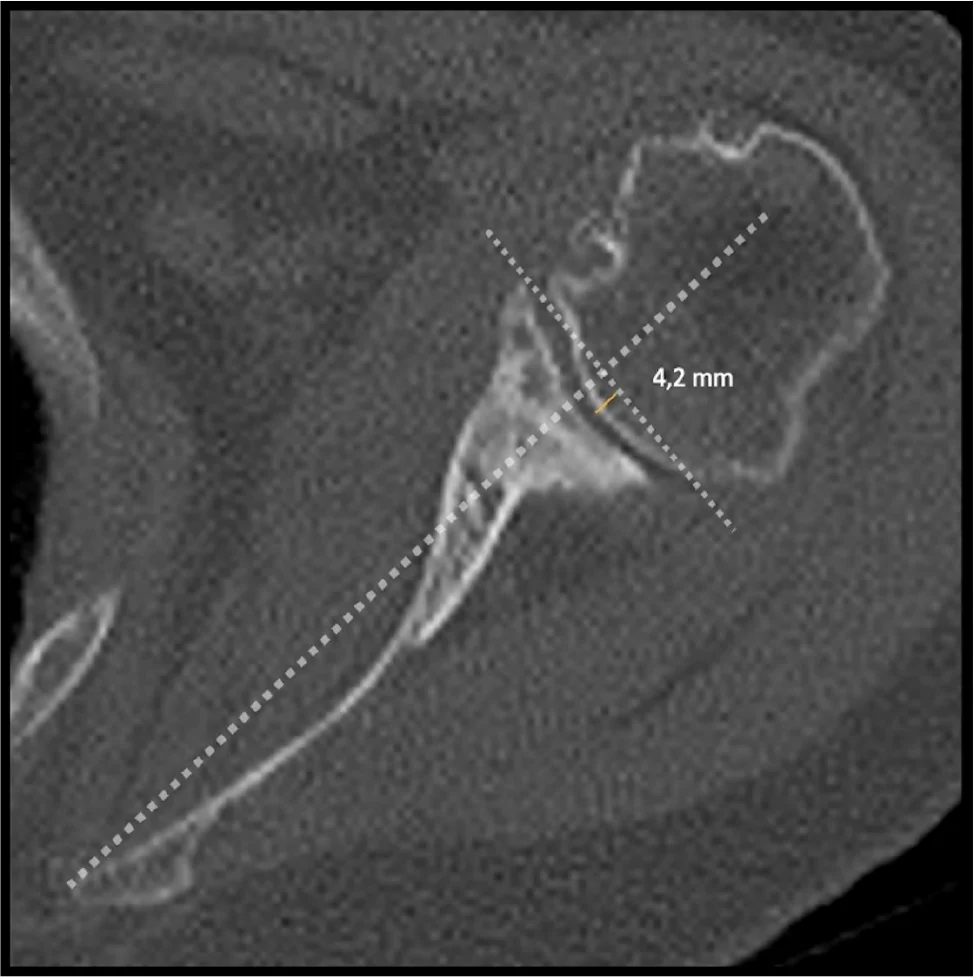
Humeral head medialization, measured by the intrusion of the humeral head medial to the premorbid glenoid line (paleogle-noid line), using axial CT images.

Humeral head medialization evaluates the degree of joint line displacement resulting from advanced osteoarthritis (Figure 15). Significant medialization often indicates severe cartilage and bone loss, limiting the surgical options^**(^43^,^51^)**^.

### Walch classification

**Figure 16:**
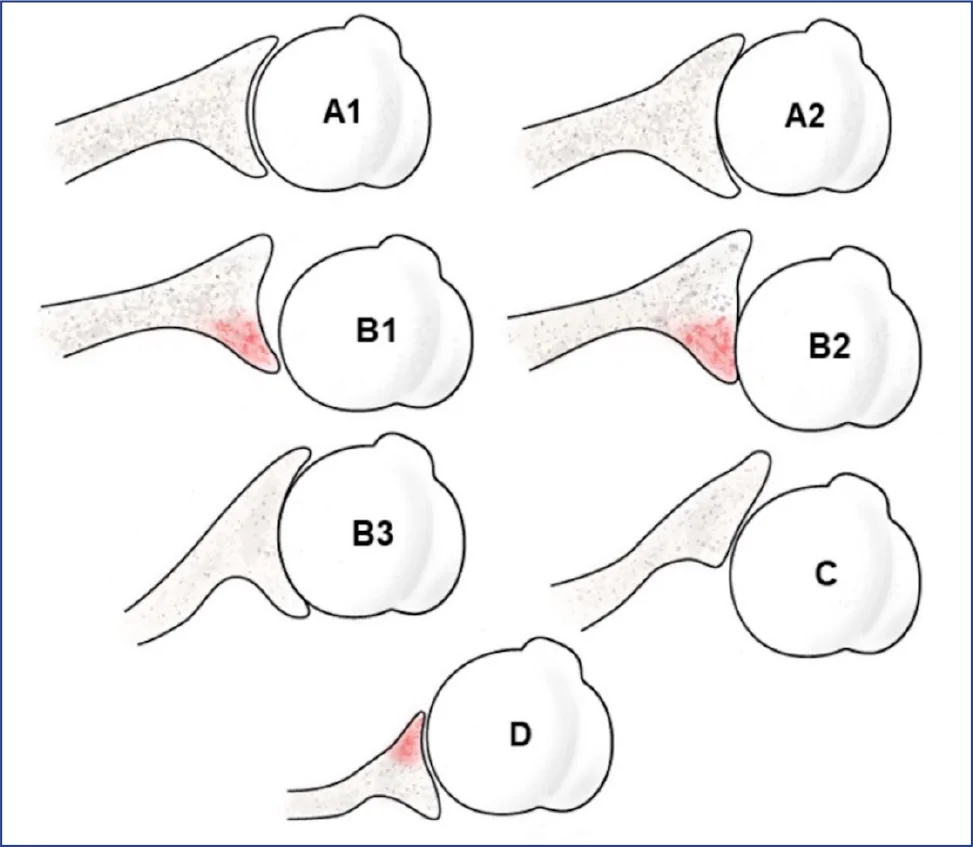
The Walch classification for glenoid morphology. There are four basic glenohumeral types based on glenoid morphology.

The Walch classification (Table 3 and Figure 16) provides a systematic approach to categorizing glenoid morphology in osteoarthritis, with important implica-tions for surgical planning and prognosis. Introduced in 1999, it remains the most widely used system to describe glenoid pathology in the context of shoulder replacement, helping guide decisions on prosthetic dep>sign, reaming, and bone grafting^**(^4^,^6^,^60^-^62^)**^. Walch type A represents symmetric glenoid morphology with no pos-terior subluxation. Due to symmetric load distribution in this glenoid morphology, there is adequate stability for joint replacement purposes^**(^4^,^6^,^43^,^60^)**^. Walch type B is common and represents asymmetric glenoid morphol-ogy, with predominant posterior glenoid wear related to progressive posterior humeral head subluxation. In this configuration, load distribution imbalances may predispose to prosthetic failure^**(^4^,^43^,^60^)**^. Walch type C is defined by accentuated glenoid retroversion, typically associated with dysplasia rather than erosion. This configuration is often congenital and predisposes the joint to severe dysfunction^**(^6^,^43^)**^.

**Table 3:** Walch classification for glenoid morphology.

Type	Description
A	Humeral head centered on the glenoid fossa
	• A1: minor central erosion
	• A2: major central erosion
B	Posterior subluxation of the humeral head
	• B1: narrowing of the posterior joint space, subchondral sclerosis, osteophytes
	• B2: biconcave aspect of the glenoid with posterior rim erosion and retroverted glenoid
	• B3: monoconcave and posterior wear with >15° retroversion or >70% posterior humeral head subluxation, or both
C	Dysplastic glenoid
	• C1: dysplastic glenoid with >25° retroversion regardless of the erosion
	• C2: biconcave, posterior bone loss, posterior translation of the humeral head
D	Glenoid anteversion or anterior humeral head subluxation < 40°

Adapted from Bercik et al.**^(^63^)^**.

Last, Walch type D represents pathologic antever-sion, frequently accompanied by anterior subluxation of the humeral head. Although less common, this subtype poses unique challenges for surgical correction^**(^43^,^61^)**^.

Advanced imaging techniques, particularly 3D CT reconstructions, have significantly improved the reproducibility of the Walch classification. Studies show better interobserver and intraobserver agreement when using 3D imaging compared with traditional CT scans, emphasizing its importance in preoperative planning. In addition, the classification provides critical insights into the relationship between glenoid morphology and joint biomechanics, particularly its association with muscle fatty infiltration and outcomes in TSA^**(^6^,^64^)**^.

### Muscle status assessment

**Figure 17:**
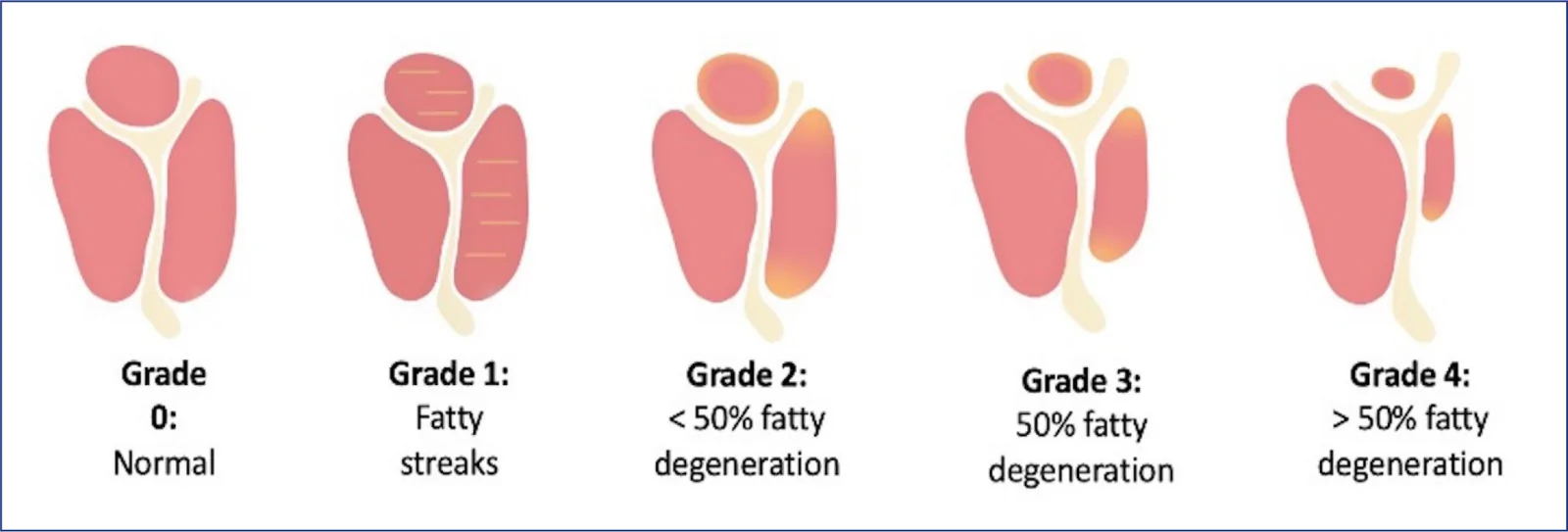
The Goutallier classification. Grade 0: no muscle fat infiltration in the rotator cuff muscles; grade 1: minimal or mild fat infiltra-tion of the muscle; grade 2: less than 50% of the muscle is infiltrated by fat; grade 3: 50% fatty degeneration; grade 4: fat infiltration ex-ceeds preserved muscle fibers in volume. As fat infiltration progresses, there is concomitant muscle atrophy.

The condition of periarticular muscles, especially the rotator cuff, is critical in determining treatment outcomes for glenohumeral osteoarthritis^**(^41^,^43^)**^. The Goutallier grading system, as depicted in Figure 17, is particularly valuable in preoperative planning, allow-ing qualitative assessment of the rotator cuff muscles. It has been demonstrated that higher grades of fatty infiltration are strongly associated with poor surgical outcomes^**(^65^)**^. In cases of severe (grade 3 or 4) fatty degeneration, reverse shoulder arthroplasty is often required to compensate for the compromised function of the rotator cuff muscles^**(^36^,^41^)**^. Deltoid muscle tro-phism should also be described in the report, because its preservation is fundamental for good postoperative outcomes of reverse arthroplasty^**(^66^)**^.

## Conclusion

Comprehensive evaluation and management of fo-cal glenohumeral chondral lesions and glenohumeral osteoarthritis requires a multidisciplinary approach that integrates anatomical understanding, adequate imag-ing techniques and clinical expertise. Advancements in imaging technologies and regenerative joint therapies hold promise for earlier detection and more conservative management of glenohumeral osteoarthritis. As these technologies continue to evolve, they offer opportuni-ties to delay disease progression and improve long-term functional outcomes for patients.

Radiologists and orthopedic surgeons play a central role in this process by leveraging these diagnostic tools to identify key imaging parameters for optimal patient care. In addition, understanding the integration of labral degeneration, rotator cuff integrity, and secondary features like synovitis or deltoid muscle atrophy also improves patient selection for procedures such as TSA.

Continued research and collaboration across disci-plines will be crucial to further enhance diagnostic ac-curacy and refine surgical and nonsurgical interventions, ultimately improving quality of life for patients affected by this complex condition.

## Data Availability

Not applicable
